# Island Study Linking Aging and Neurodegenerative Disease (ISLAND) Targeting Dementia Risk Reduction: Protocol for a Prospective Web-Based Cohort Study

**DOI:** 10.2196/34688

**Published:** 2022-03-01

**Authors:** Larissa Bartlett, Kathleen Doherty, Maree Farrow, Sarang Kim, Edward Hill, Anna King, Jane Alty, Claire Eccleston, Alex Kitsos, Aidan Bindoff, James C Vickers

**Affiliations:** 1 Wicking Dementia Research and Education Centre University of Tasmania Hobart Australia

**Keywords:** public health, online, prospective research cohort, dementia, aging, older adult, neurodegenerative, modifiable risk factors, risk reduction, prevention, lifestyle and behaviors, lifestyle, behavior change, intervention, risk, cognition, blood-based dementia biomarkers, research translation

## Abstract

**Background:**

Up to 40% of incident dementia is considered attributable to behavioral and lifestyle factors. Given the current lack of medical treatments and the projected increase in dementia prevalence, a focus on prevention through risk reduction is needed.

**Objective:**

We aim to increase dementia risk knowledge and promote changes in dementia risk behaviors at individual and population levels.

**Methods:**

The Island Study Linking Aging and Neurodegenerative Disease (ISLAND) is a long-term prospective, web-based cohort study with nested interventions that will be conducted over a 10-year period. Target participants (n=10,000) reside in Tasmania and are aged 50 years or over. Survey data on knowledge, attitudes, and behaviors related to modifiable dementia risk factors will be collected annually. After each survey wave, participants will be provided with a personalized dementia risk profile containing guidelines for reducing risk across 9 behavioral and lifestyle domains and with opportunities to engage in educational and behavioral interventions targeting risk reduction. Survey data will be modeled longitudinally with intervention engagement indices, cognitive function indices, and blood-based biomarkers, to measure change in risk over time.

**Results:**

In the initial 12 months (October 2019 to October 2020), 6410 participants have provided baseline data. The study is ongoing.

**Conclusions:**

Recruitment targets are feasible and efforts are ongoing to achieve a representative sample. Findings will inform future public health dementia risk reduction initiatives by showing whether, when, and how dementia risk can be lowered through educational and behavioral interventions, delivered in an uncontrolled real-world context.

**International Registered Report Identifier (IRRID):**

DERR1-10.2196/34688

## Introduction

Dementia is one of the most prevalent and burdensome conditions that impact the community, and projections suggest that 152 million people will be affected worldwide by 2050 [1]. In the absence of medical treatments that halt or slow disease progression, dementia risk reduction has been identified as a priority for public health research and policy development [2-6].

Recent estimates suggest that between 40% and 48% of dementia could be prevented or delayed through dementia risk reduction interventions [3,7]. Modifiable dementia risk factors include diabetes, hypertension, high cholesterol, obesity, hearing loss, physical inactivity, smoking, excessive alcohol consumption, low educational attainment, lack of cognitive stimulation, social isolation, brain injury, pollution exposure, stress, and depression [7,8]. The population attributable fraction of risk (which combines the relative risk with prevalence) for independent risk factors ranges from 1% (obesity, diabetes) to 8% (hearing loss) [7]. Behavioral and lifestyle factors can co-occur and the interplay between multiple risk factors is not yet well understood [9,10]. Models with Australian data that account for nonindependence suggest that reducing the rate of each risk factor by 20% could reduce the prevalence of dementia by up to 30% by 2050 [3].

The effects of behavioral interventions for reducing dementia risk—a group-based intervention targeting diet and exercise, with cognitive training and vascular risk factor management [11]; dietary advice [12,13]; changes in physical activity [14,15]; cognitive training [16,17]; undertaking formal education in later life [18]; and a multicomponent program targeting cognitive activity, exercise, diet, sleep, and depression management [19]—are actively being investigated. These trials [11-19] have a necessary focus on internal validity and are subject to time constraints; therefore, they largely target at-risk older individuals to maximize the chance of observing change in dementia-related outcomes within the study period. Although trial results can provide evidence of cause and effect, external validity (including the extent to which findings can be replicated in real-world settings and generalized to public health or long-term benefit) needs to be verified [11].

There has also been a concerted effort in dementia research focused on understanding the biological and functional changes that precede clinical symptoms of neurodegeneration. Promising new molecular markers that support the potential for individual dementia risk profiling have been identified [9,20]. For example, apolipoprotein epsilon-E and polygenic risk can now be identified within families, and blood-based biomarkers (eg, beta-amyloid, phosphorylated tau, and neurofilament light) can indicate neuropathology and neurodegeneration up to 20 years before functional symptoms occur [21,22]. Similarly, new web-based tools are emerging that use computer-based audiovisual techniques and artificial intelligence to remotely measure cognitive, visual, speech, and motor functions [23]. Subtle changes in thinking and memory and in lexicosyntactic and motor domains are understood to be associated with early-stage dementia [24-26]. Using these emerging techniques, it is possible to detect and track preclinical signals of neuropathology.

Life-course modeling suggests that the future incidence of dementia could be reduced by modifying dementia risk-related behaviors in midlife [7,27], and it is now important to test these models in real-world contexts. Long-term large-sample prospective cohort studies present a viable approach for tracking changes in dementia risk factors over time at the individual and population level [2,5,28]. Linking biological and functional markers of dementia pathology with information about dementia risk behaviors and demographic data provides the opportunity for detailed cohort characterization and enables precision in selecting and targeting risk reduction interventions [9]. It is then feasible to investigate patterns of change in relation to intervention engagement for symptomatic, high-risk, and asymptomatic participants. A large prospective research cohort can thus provide a framework for investigating the modification potential of individual and population-level dementia risk profiles in relation to intervention exposures and can help determine the conditions in which public health dementia risk reduction interventions could be most beneficial [29,30].

Tasmania has a population of 0.5 million that has an aging profile and higher incidences of many dementia risk factors than the rest of Australia. Furthermore, while there is a public appetite for dementia prevention, only 30% of Australians reportedly recognize that dementia risk is modifiable [31,32]. Emerging evidence indicates help-seeking older adults find that knowing their own risk profile information improves their understanding of how they can reduce their risk of developing dementia [33]. This is encouraging and offers support for providing personalized feedback to help increase the personal resonance of risk reduction messages and to stimulate health behavior change [34,35]. This knowledge, understanding that it is possible to change one’s health status, having access to the opportunity and support required to take action, and being motivated to do so are all considered to be important contributing factors for achieving effective health-related behavior change [36,37].

The primary hypothesis for this study is that increasing knowledge and motivations related to modifiable dementia risk, and engaging in behavioral and lifestyle-related risk reduction activities, will be protective over time against declining cognition and neurobiological markers of dementia, at the individual level and the population level. There are 3 key aims: (1) characterize dementia risk knowledge and behaviors, current and historical health status, cognition, and dementia-related biomarkers; (2) deliver health-related community-based and educational dementia risk reduction interventions; and (3) determine the real-world conditions in which dementia risk reduction interventions yield the greatest benefit to inform future dementia prevention research, policy, and practice.

## Methods

### Overview

The Island Study Linking Aging and Neurodegenerative Disease (ISLAND) is a large-sample prospective public health cohort study with nested interventions. Targeting dementia risk-related behaviors and modifiable dementia risk factors, ISLAND is being conducted in Tasmania, Australia.

### Ethics

The study has been approved by University of Tasmania’s Health and Medical Human Research Ethics Committee (HREC H001864) and will be conducted in accordance with the National Health and Medical Research Council’s National Statement on Ethical Conduct in Human Research [38]. Potential participants will be given up-to-date information and asked to provide consent to the research conditions at baseline; enrolled participants will be provided updated study materials and asked to reconsent at all subsequent assessment waves.

### Study Design

**Figure 1:**
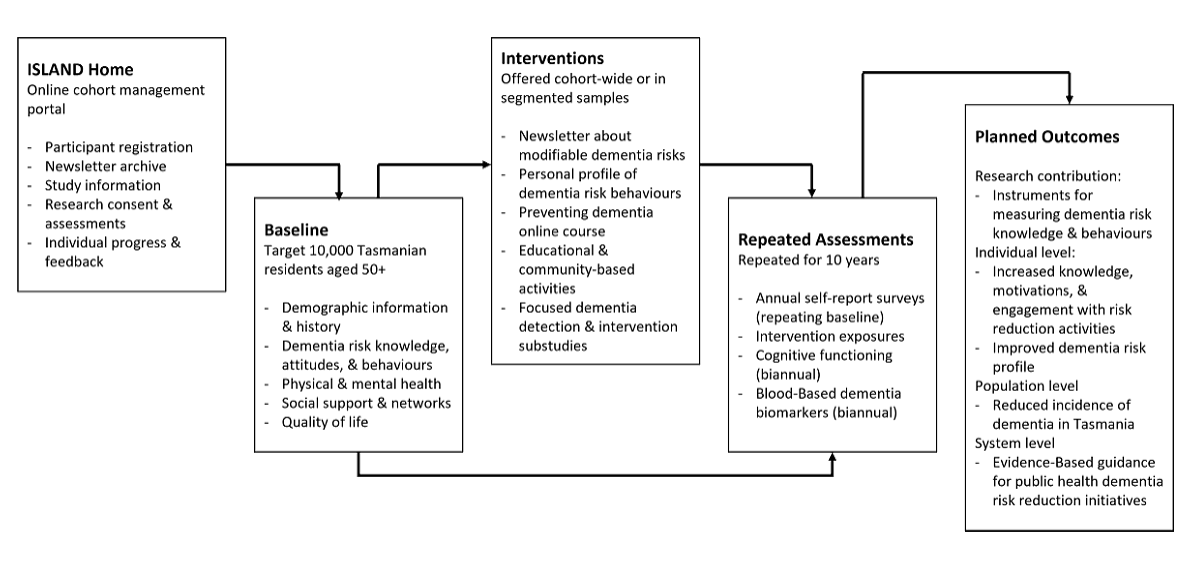
Study design and planned outcomes. ISLAND: Island Study Linking Aging and Neurodegenerative Disease.

This study will be conducted over a period of 10 years (2019 to 2029), with repeated measurements and a range of risk reduction interventions (Figure 1). We anticipate that this timeframe will be sufficient for changes in cognitive and biological markers to occur. Participants are considered active agents who are provided the opportunity to engage in managing their own dementia risk based on their knowledge, personal motivations, and circumstances, and to encourage dementia risk reduction in their communities. Longitudinal patterns of knowledge about modifiable dementia risks, motivations to engage with risk-reduction interventions, and changes in dementia risk behaviors, cognitive function, and blood-based biomarkers will be investigated. Data will be modeled to test whether, when, and how dementia risk reduction interventions are likely to reduce future dementia incidence in the long term.

The study will primarily be web-based to maximize access for Tasmanian residents regardless of their location. A dedicated, secure web-based environment has been designed to manage participant engagement and to remotely administer questionnaires and cognitive assessments. In-person attendance is only required for blood sample collection, which will take place in community-based clinics.

### Measures

Participants’ knowledge of and motivations to change behaviors related to dementia risk will be collected annually with surveys, in conjunction with current behaviors and engagement indices (Table 1). The Checklist for Reporting the Results of Internet e-Surveys [39] is available in Multimedia Appendix 1. Data from the Knowledge of Dementia Risk Reduction instrument, the Motivations to Change Lifestyle and Health Behaviours for Dementia Risk Reduction scale [40], and dementia risk profile will be used to support behavior change modeling [41]. A portion of the cohort will also complete the New General Self-Efficacy scale [42], the All Aspects of Health Literacy Scale [43], and the Perceived Stress Scale [44].

The Cambridge Neuropsychological Test Automated Battery [45,46] will be used to measure changes in thinking and memory, and Talk2Me [23], an automated linguistic data collection instrument, will be used to detect lexicosyntactic indicators of cognitive function. Motor–cognition will be measured using a screening instrument for preclinical Alzheimer disease (TAS Test) [47] that applies computer vision to capture the speed and rhythm of hand movements, which are understood to decline with the onset of dementia pathology [26]. The Cambridge Neuropsychological Test Automated Battery, Talk2Me, and TAS Test instruments are designed for web-based administration, allowing a wider reach than that which could be achieved with in-person assessment. Multisource cognitive data provide the opportunity to understand performance changes in multiple domains and can be used to triangulate, and cross-validate changes observed in other data. Data on nonmodifiable risk factors, such as age, sex, and genetics (from blood samples), and procedural factors on intervention implementation, engagement, and outcomes will also collected [29,30].

**Table 1:** Measurement instruments.

Instrument or assessment	Outcome
Background and Health Survey	Detailed demographic, health, and lifestyle characteristics
Intervention engagement	System reports of course enrolment and progression and newsletter engagement; self-report intervention and community activity engagement data
Knowledge of Dementia Risk Reduction Survey	Knowledge of dementia risk; recall and recognition of modifiable and nonmodifiable dementia risk factors
Motivation to Change Lifestyle and Health Behaviours for Dementia Risk Reduction Scale [40]	Beliefs and attitudes toward lifestyle and behavioral changes for dementia risk reduction
All Aspects of Health Literacy Scale^a^ [43]	Functional, communicative, and critical health literacy
New General Self-Efficacy Scale^a^ [42]	Perceived ability to achieve a range of different types of task.
Dementia Risk Profile	Behaviors affecting dementia risk factors: diagnosis, regular checks and management of cardiometabolic health; BMI; physical and cognitive activity; diet, alcohol consumption, and smoking
Hospital Anxiety and Depression Scale [48]	Symptom severity for 2 dimensions (anxiety and depression); pooled score indicates psychological distress
Perceived Stress Scale^a^ [44]	Extent to which daily life is perceived as stressful
Lubben Social Network Scale [49]	Extent of social networks; 3 dimensions (family, neighbors and friends)
Assessment of Quality of Life [50]	Multiattribute utility instrument that generates psychometric and Quality of Life Years scores based on 8 dimensions of health-related quality of life: physical health (independent living, pain, and senses) and psychosocial health (mental health, happiness, coping, relationships, and self-worth)
Written reflection task [51]	Cognitive performance: idea density, grammar, and sentence construction
Talk2Me Online [23]	Cognitive performance: image naming, picture description, and audio files providing approximately 2000 lexicosyntactic, acoustic, and semantic features for analysis
Cambridge Neuropsychological Test Automated Battery Online [45]	Cognitive performance: Paired Associates Learning captures learning and recall of visual information over successive trials and is sensitive to cognitive decline in early Alzheimer disease and mild cognitive impairment [52] Spatial Working Memory assesses executive function, retention, and manipulation of visuospatial information
TAS Test [47]	Motor and cognitive performance using keyboard tapping, visuomotor reaction tests, and visuospatial working memory tests providing approximately 1000 motor-cognitive features for analysis
Blood samples	Biomarker levels indicative of dementia pathology (eg, beta-amyloid, phosphorylated tau, and neurofilament light) measured in plasma or serum using enzyme-linked immunoassay, SIMOA,^b^ and mass spectrometry
Genetics	Candidate gene markers related to Alzheimer disease such as apolipoprotein epsilon-E and the brain-derived neurotrophic factor polymorphism measured via blood samples

^a^Instruments only administered to some participants.

^b^SIMOA: Single Molecule Array.

The dementia risk profile questionnaire and scoring are based on risk criteria outlined by the World Health Organization [6] and the Australian National University’s Alzheimer Risk Index [53]. For cognitive activity, the frequencies of 11 different cognitive and cultural activities are recorded (low risk: 33 or higher; high risk: less than 33). For physical activity, minutes per week of light, moderate, and vigorous activity are recorded, with each minute assigned 3.3 MET, 4 MET, and 8 MET and summed for the total score (low risk: 600 or more; high risk: less than 600). For alcohol consumption, the number of standard drinks per week is recorded (low risk: less than 14 drinks per week, and no more than 2 standard drinks per day; medium risk: 14 or less standard drinks per week and either more than 2 standard drinks per occasion or binge drinking; high risk: more than 14 standard drinks per week). Risk levels associated with blood pressure management are based on whether hypertension has been diagnosed, check-up frequency, and treatment (low risk: no diagnosis and regular check-ups, or diagnosis under medical management; medium risk: regular check-ups but with either unsure diagnosis or with hypertension and being treated; high risk: hypertension diagnosis and no regular check-ups or medical treatment, or no diagnosis and no regular check-ups). Risk levels for cholesterol management and diabetes management are scored in the same way as hypertension. BMI is calculated as weight divided by height squared (low risk: 18 to 24.9 kg/m^2^; medium risk: 25 to 29.9 kg/m^2^ or less than 18 kg/m^2^; high risk: 30 kg/m^2^ or over). Adherence to the Mediterranean-DASH Intervention for Neurodegenerative Delay diet is calculated using the total score, which ranges from 0 to 14 (low risk: 12 or higher; medium risk: 7.5 to 11.9; high risk: less than 7.5). For smoking, low risk is assigned when no smoking is reported, medium risk is assigned when occasional smoking is reported, and high risk is assigned when participants smoke daily, almost daily, or weekly. In addition to dementia risk profile scores, risk levels for depression are computed using data from the Hospital Anxiety and Depression Scale [48]. Normative cut points are applied to assign risk levels: low (normal, 0 to 7), medium (borderline, 8 to 10) and high risk (11 to 21).

### Setting and Participants

Tasmania’s population is distributed across urban, rural, and remote settings. A larger percentage of residents (19.4%) are over the age of 65 years (compared to 14.8% nationally), and Tasmania has the highest median age of all Australian states (Tasmania: 42 years; Australia: 37 years) [54]. Relative to the Australian population, Tasmania has high rates of smoking (Tasmania: 16.4%; Australia: 13.8%); obesity (Tasmania: 70.9%; Australia: 67.0%); heart, stroke, or vascular disease (Tasmania: 6.0%, Australia: 4.8%); diabetes (Tasmania: 5.5%, Australia: 4.9%); and poor mental health (Tasmania: 21.7%, Australia: 20.1%). Educational attainment is also low in Tasmania, with 27.5% of the population aged 15 to 75 years having no educational qualification above year 10 (or 16 years of age) compared with the national average of 19.3% [55]. These characteristics mean the Tasmanian population is at higher risk of dementia than the broader Australian population and is ideal for a public health research initiative implementing strategies to achieve dementia risk reduction.

### Recruitment and Retention

We aim to recruit 10,000 participants, which is 5% of the Tasmanian population aged 50 or over. This sample offers a meaningful slice of the population for the project’s public health objectives. Eligibility criteria are age 50 years or older (based on the likelihood of higher personal health motivations and to provide maximum opportunity for observing longitudinal changes from midlife in behavioral, cognitive, and biological markers [35]), resident of Tasmania, having an email address, and having access to the internet. Invitations to join the study are regularly and widely distributed using web-based, print, and broadcast media; community talks; information booths; social media; flyers; and posters. Potential participants register interest via ISLAND Home, a secure web-based cohort management portal [56]. On their personal ISLAND Home page, participants can monitor their study engagement history and progress, update their contact information, access newsletter archives, and respond to research and intervention invitations. ISLAND project staff liaise with participants by email, through social media, and in person at the community-level to support recruitment, engagement, and retention. Participants who self-identify as having deteriorating cognitive functioning in their annual survey responses will be advised to seek medical referral to the affiliated ISLAND Clinic for clinical assessment, diagnosis, and treatment.

### Interventions

#### ISLAND Newsletter

Upon registration, participants are sent an email newsletter every 3 to 4 weeks. The newsletters provide a digest of evidence-based information, project findings, information about modifiable dementia risks, and invitations to join research and community activities.

#### Dementia Risk Profile

Participants complete a dementia risk profile questionnaire at each timepoint. Upon completion, they are provided with a personalized report of their individual risk level (low, medium or high) for 9 behavioral and lifestyle domains, and includes advice, based on World Health Organization guidelines [6], for shifting to a lower risk level. The dementia risk profile is intended to be used by participants to monitor changes in their own dementia risk-related behaviors over time, as a source of motivation for addressing risk, or to share with their primary health provider for guidance in risk factor management.

#### Preventing Dementia Massive Open Online Course

The Preventing Dementia Massive Open Online Course presents contemporary evidence-based information on dementia risk and guidance on risk reduction behaviors [57]. The free 4-week course is offered twice each year. Quizzes provide participant feedback, and social engagement is supported by monitored discussion forums. In 2019, the Preventing Dementia Massive Open Online Course was ranked by Class Central as the all-time third highest among health-related Massive Open Online Courses [58]. Since its launch in 2016, the course has attracted 154,000 enrolments from 175 countries, of whom 43% have completed the course. Based on pilot evidence [59], exposure to the course is expected to increase knowledge and motivations related to modifiable dementia risks, and thus support changes in dementia risk behaviors.

#### ISLAND Campus

Evidence from the Tasmanian Healthy Brain Project [60,61] showed later life university education may protect against cognitive decline. Between June and September 2020, full fee-waiver scholarships were made available by the University of Tasmania to study participants to undertake a full university course of their choosing. To be eligible, participants needed to provide additional survey data (Perceived Stress Scale, New General Self-Efficacy Scale, All Aspects of Health Literacy Scale). These data will be used to measure the influence of perceived stress, self-efficacy, and health literacy on student engagement, and the combined long-term effect of university study in later life on dementia risk knowledge, attitudes, behaviors, and cognitive functioning will be examined.

We have also put in place partnerships with established government and nongovernment health promotion organizations (eg, Tasmanian Health Service, Libraries Tasmania, municipal offices within the Local Government Association of Tasmania, Quit Tasmania, The Heart Foundation, Diabetes Tasmania). These partnerships will be leveraged to provide study participants opportunities to connect with local community-based public health activities that target dementia risk factors.

#### Future Interventions

This study is a valuable opportunity to conduct substudies focusing on specific research questions. For example, cohort segments can be targeted with particular interventions, or recruited into studies of factors that influence or detect change in dementia trajectories. Participant invitations to join future studies, health promotion programs, and community-initiated events (walking groups, information sessions, social groups) will be promulgated via notices on ISLAND Home, in newsletters, and on social media. Biological samples collected from participants, such as blood, will support further investigation of links between proteins, genetic markers, neurodegenerative disease, and brain function.

### Data Management and Storage

Survey, cognition, and biomarker data will be deidentified and stored on secure databases in accordance with institutional ethics and privacy policies. Blood samples will be stored indefinitely in secure freezers (at –80 °C) for future analyses.

Data is collated using the secure web-based portal. Survey, cognitive assessment, biomarker, and intervention data are linked by a unique identifier, forming a comprehensive database of participant demographics, risks, exposures, functioning, neuropathology, and potential confounders at participant level.

### Planned Analysis

Relevant literature will be monitored to inform covariate selection and exclusion criteria. Missing data mechanisms will be assessed and accounted for using standard techniques.

Multilevel regression models will be used to estimate changes in dementia risk knowledge, attitudes and behaviors over time with respect to exposures. These models allow control of type 1 errors where data do not meet assumptions of independence. Multinomial state-space models will be used to model changes in risk behaviors over time by estimating the expected probability of transition from state *i* at time *t–*1 to state *j* at time *t*, where states *i* and *j* represent discrete risk (eg, high risk and low risk). Longitudinal profiles of cognitive assessment and biomarker trajectories will be modeled using multilevel regression models, adjusting for covariates including age, sex, years of education, and intervention exposure. Multivariate relationships between risk knowledge, attitudes, and behavior states with dementia risk reduction, cognitive assessments, biomarkers, measured exposures, other demographic data, and survey data will be explored using clustering or ordination techniques such as *t*-distributed stochastic neighbor embedding.

## Results

**Figure 2:**
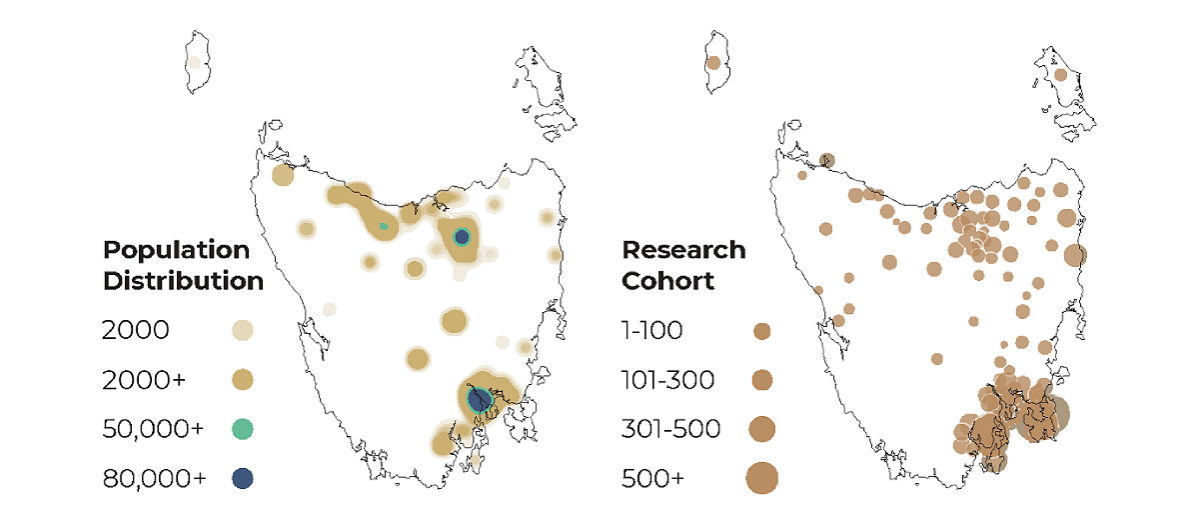
Geographical distribution of the Tasmanian population (N=542,000) and study participants (per 100, n=6410). The map was traced from Google Maps [62], and population [63] and participant data were overlaid using Google’s JavaScript and Geocoding APIs.

Recruitment in the first 12 months (October 2019-October 2020) indicates the target baseline sample is feasible. As of October 1, 2020, a total 12,706 people had registered interest in the study, of whom 6410 provided complete data in the baseline demographic and dementia risk profile surveys. The cohort is distributed across the state’s populated regions, with greater density in the capital city (south) and regional urban centers (north and northwest) (Figure 2). The characteristics of the ISLAND cohort compared with the Tasmanian population are presented in Table 2. Study participants’ median age is 63 years, and there is a nearly even split between retired (3002/6410, 46.8%) and employed (2924/6410, 45.6%) participants. Nearly three-quarters (4630/6410, 72.2%) are female, and a higher proportion have university degrees (3107/6410, 50.0%) than population norms (36,337/206,421, 17.5%). There is also a higher proportion of people living in areas of socioeconomic advantage (857/6410, 13.4%) than in the general population (9495/206,421, 4.6%). Of 6410 participants, 1731 (27.0%) have completed the Preventing Dementia Massive Open Online Course. Recruitment is complete for ISLAND Campus; 1322 participants are enrolled, of whom 784 (59.3%) have commenced a new university course.

**Table 2:** Study participants (October 2019-October 2020) compared with Tasmanian residents 50 years and older. Tasmanian population data drawn from the Australian Bureau of Statistics [63].

Demographic variables		Participants (n=6410), n (%)	Tasmanian population >50 years of age (n=206,421), n (%)
**Age (years)**			
	Mean (SD)	63.1 (7.5)	65.3 (10.6)
	Median (range)	63 (50, 94)	63 (50, 105)
**Age categories (%)**			
	50-59 years	2306 (36.0)	72,912 (35.3)
	60-69 years	2724 (42.5)	67,723 (32.8)
	70-79 years	1237 (19.3)	42,044 (20.4)
	80+ years	143 (2.2)	23,742 (11.5)
**Gender (%)**			
	Female	4630 (72.2)	108,014 (52.3)
	Male	1771 (27.6)	98,418 (47.7)
	Other	9 (0.01)	N/A^a^
**Work Status (%)**			
	Retired	3002 (46.8)	118,221 (49.1)
	Employed/Work-ready	2924 (45.6)	105,931 (41.9)
	Missing	78 (0.01)	16,816 (0.07)
**Education level (%)**			
	Postgraduate degree	1832 (28.6)	12,344 (5.9)
	Bachelor's degree	1275 (21.5)	23,993 (11.6)
	Diploma/trade	1929 (30.1)	62,095 (34.4)
	High school	1018 (15.9)	52,192 (28.9)
	Primary school	4 (0.01)	29,756 (16.5)
	Missing	252 (3.9)	N/A
**Regional distribution (%)**			
	North and northeast	1452 (22.7)	58,094 (28.2)
	West and northwest	919 (14.3)	46,597 (22.6)
	South and southeast	4006 (62.5)	101,456 (49.2)
	Missing	27 (0.4)	0 (0)
**Socioeconomic status^b^ (%)**			
	Quintile 1	1822 (28.4)	76,788 (37.2)
	Quintile 2	1380 (21.5)	53,876 (26.1)
	Quintile 3	914 (14.3)	37,775 (18.3)
	Quintile 4	1404 (21.9)	28,486 (13.8)
	Quintile 5	857 (13.4)	9495 (4.6)
	Missing	33 (0.5)	0 (0)

^a^N/A: not available.

^b^Quintile 1 is the most relatively disadvantaged areas, and Quintile 5 is the most relatively advantaged areas.

**Figure 3:**
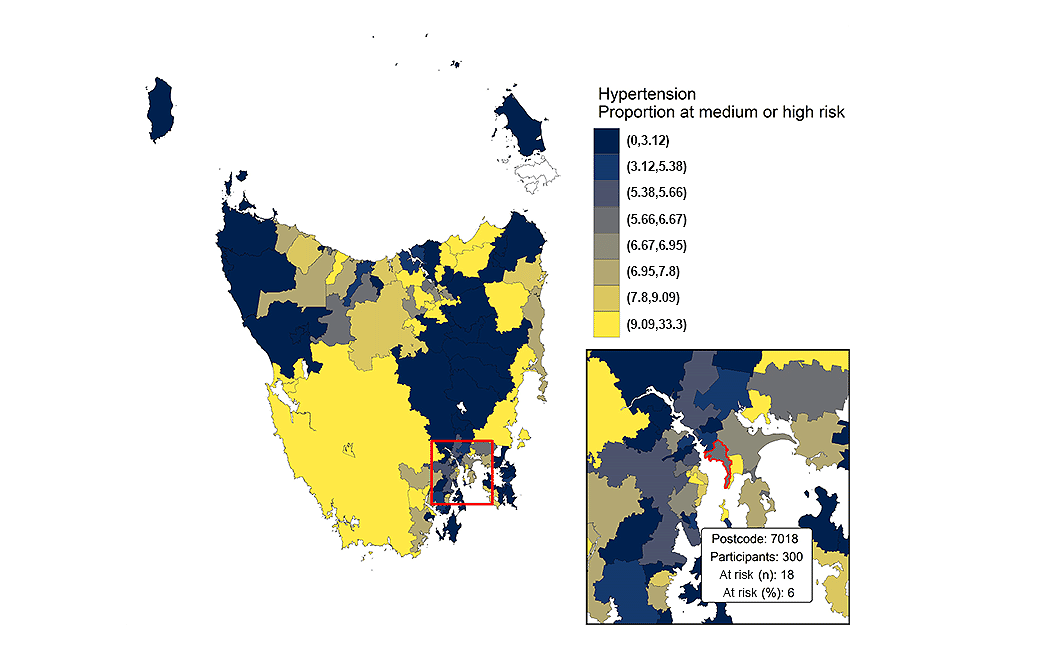
Participants at high or medium risk due to unmanaged hypertension, by postcode. The map was created using ggplot2 in R [64] overlaid with 2016 postcode boundaries from the Australian Bureau of Statistics [63].

Risk status for 10 of the established modifiable dementia risk factors [7,8] (Table 3) and the geographical distribution of participants scoring high risk for unmanaged hypertension (Figure 3) are shown. Figure 3 is presented to show how the combination of dementia risk profile data and geographical location enable the identification of areas where community-based risk reduction interventions might be most warranted.

**Table 3:** Risk status of participants for 10 modifiable dementia risk factors.

Risk factor and level		Participants (n=6410), n (%)
**Cognitive activity**		
	Low	3519 (54.9)
	High	2800 (43.7)
**Physical activity**		
	Low	5429 (84.7)
	High	741 (11.6)
**Alcohol consumption**		
	Low	3207 (50.0)
	Medium	1934 (30.2)
	High	779 (12.1)
**Blood pressure**		
	Low	5925 (92.4)
	Medium	175 (2.7)
	High	258 (4.0)
**BMI**		
	Low	2293 (35.8)
	Medium	2197 (34.3)
	High	1616 (25.2)
**Cholesterol level**		
	Low	5209 (81.3)
	Medium	230 (3.6)
	High	922 (14.4)
**Diabetes**		
	Low	5270 (82.2)
	Medium	113 (1.8)
	High	963 (15.0)
**Mediterranean diet adherence**		
	Low	1284 (20.0)
	Medium	4646 (72.5)
	High risk	435 (6.8)
**Smoking**		
	Low	6109 (95.3)
	Medium	80 (1.2)
	High	205 (3.2)
**Depression**		
	Low	5784 (91.1)
	Medium	407 (6.4)
	High	155 (2.4)

## Discussion

This project presents a unique opportunity to observe, at individual and population levels, the long-term natural life-course trajectories related to dementia indicators and risk behaviors and responds to calls for public health action on dementia prevention [2]. The overall objective of the project is to extend and translate epidemiological and experimental intervention evidence regarding modifiable dementia risk factors, integrate life-course perspectives and inform policy development for public health dementia risk reduction initiatives [9,10]. By including 10,000 participants (or 5% of the target population aged 50 years or over), characterizing dementia risk at individual and aggregate levels, and tracking changes in relation to engagement with risk reduction interventions, this study will contribute valuable knowledge about the long-term effects of adjusting health behaviors in middle and later life on dementia risk [6,28]. This is a valuable opportunity to find out whether, when, and how dementia risk can be lowered through risk reduction interventions delivered in an uncontrolled population-based public health context.

We will apply and develop innovative web-based techniques for remotely assessing cognitive function and at scale. These functional data will be combined with detailed demographic data and data on established and emerging biomarker and genetic indicators, to characterize dementia risk in this population cohort. The research sample can thus be segmented for interventions and research questions (eg, ISLAND Campus) as well as broad-scale offerings (eg, Preventing Dementia Massive Open Online Course). Even with anticipated attrition that is common in longitudinal cohorts [65], the large sample size provides the opportunity to identify patterns of change in dementia risk over time, accommodating a wide range of behavioral and health-related antecedents, procedural factors, and demographic covariates.

While our target population sample is an ambitious goal, our preliminary results indicate that it is achievable. The early responder sample has a high proportion of women and people with university qualifications, and the proportions at medium or high risk of dementia due to obesity, smoking, and depression are lower than those in the Tasmanian population [54]. This means our sample is not yet reflective of Tasmanian norms. Because educational attainment is an established determinant of socioeconomic inequalities in both physical health and cognitive functioning [66], we expect people with lower levels of education and living in areas of relative socioeconomic disadvantage may have poorer health profiles and stand to benefit more from engaging with a dementia risk reduction public health program. Ideally, we aim to achieve a representative research sample, to maximize the reach of risk reduction information and opportunities and to support the future generalization of findings. The demographic profile of the current sample will guide targeted recruitment calls to increase the number of men and people with low formal education. Ongoing efforts are focused on recruitment through community-based networks in lower socioeconomic status areas, facilitated by partner organizations such as information technology access centers, neighborhood houses, municipal offices, and technical or trade-dominant workplaces. We anticipate that this targeted and supported recruitment strategy will increase the representation of people who may not otherwise join a web-based research program and whose data will contribute to more population-equivalent distributions across the risk domains (eg, smoking, BMI, diet). A related study [67] is examining the drivers of reach and resonance of risk reduction messages at community and population level and will inform the ongoing development and dissemination of actionable information about dementia risk reduction. Cultural and linguistic diversity was not included in the initial baseline data but is increasingly recognized as an important determinant of health and health behaviors. These data are being collected from the sample in follow-up surveys.

Because we use a participant-centered design that is informed by behavior change theory and public health research methods, participants are not required to join predetermined research interventions, they are invited to choose from a range of opportunities. The choices that participants make are based on their own knowledge, beliefs, attitudes, and circumstances. In the absence of existing measures that can be used to model the role of knowledge in behavior changes, data from this project will support the validation of new research instruments that assess dementia risk reduction knowledge (Knowledge of Dementia Risk Reduction instrument) and dementia risk behaviors. The constructs measured by these instruments, along with dementia risk reduction motivations and attitudes [40], health literacy [43], and self-efficacy [42], are central to understanding behavior change in relation to dementia risk. Initial development of these instruments was conducted using data samples separate from ISLAND data. The TAS Test motor–cognitive screening test is currently being validated with drawn from within the ISLAND sample. It is our hope that these 3 new tools will strengthen research into dementia risk reduction within and beyond the ISLAND Project. Using multisource data from the Cambridge Neuropsychological Test Automated Battery, Talk2Me, and TAS Test cognitive assessments in conjunction with blood-based biomarkers and self-report surveys will enable triangulation of objective and self-report data. This will add weight to the validity of ISLAND findings, and providing valuable insights into the potential for altering pathological dementia trajectories through behavioral intervention.

The web-based portal is intended to support the project’s reach across Tasmania’s distributed population. The framework is scalable and can be replicated for deployment in other populations, and thus offers multiple opportunities for research collaborations in the field of dementia risk reduction. Independent of their contribution to dementia, many of the modifiable dementia risk factors—particularly obesity, smoking, hypertension, depression, isolation, and diabetes—often co-occur and are directly associated with poor quality of life and mortality [68]. Collaboration at the community level with established health promotion organizations provides a sustainable and cost-effective way for participants to be alerted to risk reduction activities occurring in their area. Linking participants’ geographical location with risk profile information (Figure 3) provides the opportunity to identify areas with a high proportion of risk behaviors. This enables a data-driven rationale for partner organizations (eg, Diabetes Tasmania, Quit Tasmania) to direct community-based interventions into these areas. Thus, our approach has the potential to incidentally address other chronic health conditions and could feasibly lead to considerable public health benefits. Indeed, during the initial COVID-19 lockdown period in Tasmania, in contrast to global public narratives, ISLAND participants did not report detrimental changes in their mental or physical health [69]. The design of this study may prove to be a model that is particularly valuable for low- to middle-income countries where both dementia risk and multimorbidity are high. Ultimately, it is our intention that this project will advance what is known about behavioral and lifestyle dementia risk reduction and contribute to global public health research and practice focused on preventing dementia.

## Appendix

Multimedia Appendix 1 Checklist for Reporting Results of Internet E-Surveys.

## Abbreviations

ISLAND: Island Study Linking Aging and Neurodegenerative Disease
